# MRI acquisition and reconstruction cookbook: recipes for reproducibility, served with real-world flavour

**DOI:** 10.1007/s10334-025-01236-4

**Published:** 2025-03-06

**Authors:** Jonathan I. Tamir, Moritz Blumenthal, Jiachen Wang, Tal Oved, Efrat Shimron, Moritz Zaiss

**Affiliations:** 1 Chandra Family Department of Electrical and Computer Engineering, The University of Texas at Austin, Austin, 78712 TX USA; 2 Oden Institute for Computational Engineering and Sciences, The University of Texas at Austin, Austin, 78712 TX USA; 3 Dell Medical School Department of Diagnostic Medicine, The University of Texas at Austin, Austin, 78712 TX USA; 4 Institute of Biomedical Imaging, Graz University of Technology, Stremayrgasse 16/III, 8010 Graz, Austria; 5 Department of Biomedical Engineering, The University of Texas at Austin, Austin, 78712 TX USA; 6 Department of Electrical and Computer Engineering, Technion-Israel Institute of Technology, 3200004 Haifa, Israel; 7 Department of Biomedical Engineering, Technion-Israel Institute of Technology, 3200004 Haifa, Israel; 8 May-Blum-Dahl Technion Human MRI Research Center, Technion-Israel Institute of Technology, 3200004 Haifa, Israel; 9 Institute of Neuroradiology, University Hospital Erlangen, Erlangen, Germany; 10 Artificial Intelligence in biomedical engineering (AIBE), Friedrich-Alexander University Erlangen-Nürnberg, Erlangen, Germany

**Keywords:** Computational MRI, Reproducible research, Acquisition, Reconstruction

## Abstract

MRI acquisition and reconstruction research has transformed into a computation-driven field. As methods become more sophisticated, compute-heavy, and data-hungry, efforts to reproduce them become more difficult. While the computational MRI research community has made great leaps toward reproducible computational science, there are few tailored guidelines or standards for users to follow. In this review article, we develop a cookbook to facilitate reproducible research for MRI acquisition and reconstruction. Like any good cookbook, we list several recipes, each providing a basic standard on how to make computational MRI research reproducible. And like cooking, we show example flavours where reproducibility may fail due to under-specification. We structure the article, so that the cookbook itself serves as an example of reproducible research by providing sequence and reconstruction definitions as well as data to reproduce the experimental results in the figures. We also propose a community-driven effort to compile an evolving list of best practices for making computational MRI research reproducible.

## Introduction

Over the last 20 years, magnetic resonance imaging (MRI) research has expanded beyond the chemical and physical sciences to *computational science*. Advances in scanner hardware, numerical simulation, and image reconstruction, together with the huge increase in computation power available on modern parallel hardware, have enabled the development of advanced MRI applications which were not possible before due to long image acquisition or reconstruction times. Yet, these advances do not come without difficulties: the complexity of software and iterative algorithm frameworks render many published works difficult to reproduce. Most exciting methods are unfortunately never tried in larger studies because of the substantial effort to re-implement them.

In the scientific community in general, and in the MRI community in particular, a major emphasis has been placed on reproducibility [1–4]. The growing trend is the result of a *reproducibility crisis*, whereby the results in a significant number of scientific publications were not successfully reproduced by independent groups following the same methodology. While there are numerous causes, the lack of availability of methods/codes is a major contributing factor, as found in a survey of 1576 researchers conducted by Nature [5].

***What is reproducibility?***

Unfortunately, reproducibility, replicability, and other similar “R-words” [6] have overloaded definitions depending on the specific scientific community [7]. We follow the 2019 consensus report of the National Academies of Sciences, Engineering and Medicine [8] in defining these terms:

**Reproducibility** is obtaining consistent results using the same input data; computational steps, methods, and code; and condition of analysis.

**Replicability** is obtaining consistent results across studies aimed at answering the same scientific question, each of which has obtained its own data.

The former definition is consistent with “computational reproducibility,” first developed by Claerbout and Karrenbach [9] and later popularized by Donoho and colleagues [10, 11]. In the context of MRI acquisition, the term “input data” can refer to the scanned object as a physical storage of the spin system, or it can refer to a digital phantom in a sequence simulation environment.

While reproducibility is paramount across all aspects of magnetic resonance techniques and their applications in medicine and biology, we argue that MRI acquisition and reconstruction research is inextricably linked to the computational tools and algorithms used for experiment and analysis. We adopt the term *computational MRI* to capture the core connection to computational science, and we focus on reproducibility in this context. Under this lens, Buckheit and Donoho summarized Claerobout and Karrenbach’s main mantra, which we reproduce here:

“An article about computational science in a scientific publication is **not** the scholarship itself, it is merely **advertising** of the scholarship. The actual scholarship is the complete software development environment and the complete set of instructions which generated the figures.”

- Buckheit and Donoho [10]

The benefit of releasing common code is evident in the computer vision and machine learning communities: the availability of public implementations has resulted in rapid growth and innovation. For example, reference implementations in a variety of programming languages are available for all previous winners of the ImageNet Large Scale Visual Recognition Challenge [12]. Communities such as Hugging Face [13] have developed common platforms to share machine learning models and datasets. The neuroimaging community has also placed a great emphasis on reproducibility [14]. Tools such as Nipype [15] facilitate building reproducible computational graphs for neuroimaging workflows (e.g., [16, 17]). Brainlife.io [18] provides access to free compute and storage resources to run data workflows and automatically capture provenance graphs. A recent comprehensive review paper on open and reproducible neuroimaging, with relevance to computational MRI, was also published [2].

***Reproducibility challenges in computational MRI***

Reproducibility and replicability of computational MRI studies are hindered by several challenges spanning acquisition, reconstruction, data, and tooling.

*Acquisition challenges*. Key obstacles to research reproducibility and applicability involve the variability of MRI acquisition protocols across systems, sites, and scans due to differences in hardware and vendor-specific implementations. Although the main parameters of pulse sequences, such as repetition time (TR), echo time (TE), and radio frequency (RF) pulse flip angles, are typically reported, substantial variability exists in how these sequences are programmed, even with identical nominal parameters. Sequence descriptions in a paper’s methods section accompanied by MRI sequence diagrams often leave out unmentioned assumptions. Hardware variations impact image acquisition and quantitative measurements, influenced by differences in field strength [19], gradient performance [20], RF transmit and receive systems [21], and spectrometer design [22]. Operator-dependent factors, such as patient positioning, coil placement, and adherence to imaging protocols, introduce further variability.

These challenges were recently demonstrated in the 2020 International Society for Magnetic Resonance in Medicine (ISMRM) reproducibility challenge, which explored whether the acquisition details provided in a seminal paper on T1 mapping were sufficient to ensure reproducibility by independent research groups [23]. Eighteen submissions were received, where T1 mapping data were obtained by scanning either the ISMRM/NIST phantom [24] or human subjects. The centralized analysis showed that the variability in T1 measurements between different groups was twice as high as the variability within individual groups, despite the use of standardized phantoms and strictly controlled conditions. The conclusion was that the original paper lacked the specificity required for consistent reproduction of quantitative MRI results.

*Reconstruction challenges*. Reproducibility in MRI reconstruction is hindered by multiple factors, including variability in reconstruction algorithms, lack of standardized preprocessing workflows, and insufficient reporting of computational methods. Challenges also arise from the lack of transparency of vendor-specific reconstruction workflows, as such pipelines are often closed and protected by intellectual property laws. Data-processing steps, e.g., image interpolation methods [25], low-pass filtering [26], intensity correction [27], and gradient nonlinearity correction [28], can impact reproducibility. Many of these elements remain proprietary and function as black-box processes; hence, researchers often receive processed data without knowing the data preprocessing pipelines [29]. Reconstruction variability was explored in the 2019 ISMRM reproducibility challenge [30], which invited teams to reproduce the results of the seminal SENSE-based image reconstruction paper [31] on data supplied by the challenge organizers. Though there was a large degree of variability due to unspecified parameters such as reconstruction field of view, the image quality across teams was relatively similar.

*Data challenges*. Sharing data can also be difficult [4]. In many cases, data come from human subjects scans and may have been performed on patients through institutional review board approval; thus, sharing the data may require additional regulatory approval. If it can be shared, there is a monetary cost to publicly hosting the data, which may recur monthly or annually. Several data platforms currently front this burden, though it is unclear if the data will be accessible in perpetuity. As computational experiments grow in complexity and involve large amounts of data, e.g., for training machine learning models, it may not be feasible to share the entire dataset. When the data are available, the publication may lack specific details on which files were used in the experiments, for example in specifying training/validation/test splits. Even when *raw* data are made available, challenges arise from the absence of standardized data formats, which complicates data sharing and analysis across different platforms. As we will show, supplying only the k-space data and trajectory may not be sufficient for reproducibility. Efforts like the ISMRM Raw Data format [32] aim to address this by providing a community-driven standard for storing and sharing raw k-space measurements and associated scan metadata.

*Tooling challenges*. Another obstacle to computational research surrounds the software environments themselves. As time passes, the underlying software can be prone to *bit rot*: certain programs or functions may stop working, even if “nothing has changed.” This is because everything else—from the operating system, to the software environment, to the hosting services—may change over time, breaking backwards-compatibility [33]. Thus, the code of a finished research project may require periodic updates to stay usable [11]. As services are increasingly monetized, it is also possible for once-reliable hosting platforms to completely disappear or have trust eroded, as was the case with SourceForge [34]. Choice of software/data license may also have implications on reuse [1, 30, 35].

***Reproducibility efforts in computational MRI***

Efforts by the computational MRI research community are also materializing, strengthened by the creation of the ISMRM Reproducible Research Study Group (RRSG). The RRSG has conducted member-initiated symposia at the 2021, 2022, and 2023 Annual Meetings of the ISMRM, as well as created initiatives such as the reproducibility team challenges [36], and the MR-Hub and MR-Pub websites, which host toolboxes and code packages of published papers, respectively [37]. The Magnetic Resonance in Medicine (MRM) journal has recently created an official mechanism for code review [38] of submitted manuscripts, which encourages authors to share their code and data, and regularly highlights publications offering open code. Furthermore, the Magnetic Resonance Materials in Physics, Biology and Medicine (MAGMA) journal requires all original research articles to include a data availability statement, and the European Society for Magnetic Resonance in Medicine and Biology (ESMRMB) “MRI together” initiative [39] fosters global collaboration on open, reproducible, and inclusive MRI research.

Open-source tools for sequence definition [21, 40–42], MRI simulation [43–49], and reconstruction [32, 50–54] are maturing and establishing stable software interfaces. These tools are being combined into open-source workflows supporting reproducible research [55]. Several working groups have developed consensus reports aimed at improving reproducibility [56–63]. Additionally, benchmarking papers offer code for many different reconstruction methods and algorithms [64, 65]. Written papers that are the cornerstone of our scientific communication can be created in a fully reproducible manner using tools like Neurolibre [66] and Jupyter Book [67].

While open-source tools are not explicitly required for reproducible research, their use is highly encouraged. This is because they allow other researchers to reuse the code and data, and ultimately build on the results. It is important to understand the licensing terms of any existing software that is used, as well as to pick a suitable license for the new research work [1, 30, 35]. When commercial intellectual property needs protection, it is still possible to select a license that allows reuse for research purposes and non-commercial use. See the following webpage for guidance on selecting an appropriate license: https://choosealicense.com.

***The idea of this cookbook: recipes for reproducibility in MRI***

**Fig. 1:**
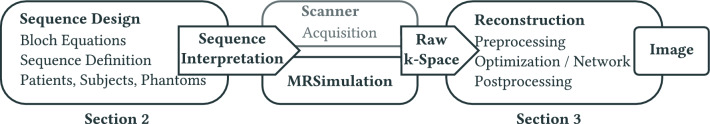
Overview: We consider reproducible MRI as a three-step pipeline of reproducible sequence design, acquisition (or simulation), and reconstruction, leading to a final image. Sequence interpretation and the raw k-space data yield interfaces between these steps. We focus on the components in black text in this article

A key step to fostering reproducibility is the development of best practices and standards to (1) get source code and data published in the first place, (2) make software available in a form which allows numerical results to be reproduced, and (3) integrate methods into other frameworks for further development or evaluation in larger experimental studies. To aid in reproducible research, we have assembled this cookbook to serve as both a guiding example for how to make computational MRI research reproducible, as well to as highlight common pitfalls when taking these recipes and trying to cook. We consider a three-step pipeline for reproducible sequence design, acquisition, and reconstruction, as shown in Fig. 1. We focus on sequence design and reconstruction, recognizing that reproducibility at the scanner level is harder to achieve, though MRI simulations can form a surrogate.

We formulate the basic descriptions of MRI acquisition and reconstruction and share recipes for their reproducibility. We showcase and describe examples where under-specification leads to markedly different results. We structure this article in such a way that it itself forms a cohesive reproducible research study. Each figure that corresponds to a computational experiment is accompanied by a specific script to reproduce it. An interactive notebook containing the computational experiments is also included.

All data, scripts, and installation instructions are shared in our corresponding GitHub repository: https://github.com/MRsources/Article_MAGMA24_RMRI.

Recognizing that the specific set of parameters that need to be specified for full reproducibility is a moving target, we propose a community-driven initiative to aggregate these parameters in the Github repository. **We invite the readers of this article to contribute to the** **issues of MRI reproducibility**^1^**to highlight experimental details that should be included to aid in reproducibility.** While we have populated common issues that we have highlighted herein, this article is by no means comprehensive. Finally, there are several excellent review papers on implementing reproducible research in adjacent communities [1, 2, 35, 68–70]; many of these recommendations directly apply to our field.

***“Good” research vs. reproducible research***

Is all reproducible research, “good” research? We emphasize that reproducibility alone is not enough—it is a necessary but insufficient condition for good science. A research result may be reproducible, but either incorrect (e.g., due to a bug in the code) or not clinically relevant (e.g., due to unrealistic assumptions). One well-documented issue in this context is the “inverse crime,” where the same forward model used to simulate data is then applied during the reconstruction process, effectively overestimating algorithm performance by neglecting real-world imperfections [71].

Computational research also often focuses on improving quantitative benchmark performance such as mean squared error. However, this can lead to visually appealing images while severely undermining their clinical utility. Evaluation metrics developed for natural images, such as structural similarity (SSIM) index [72], may fail to capture the relevant characteristics of medical images and compromise the diagnostic results [73]. Kustner et al. [74] point out that, while deep learning methods often outperform conventional ones in quantitative metrics, they may overfit to the specific application and require careful evaluation before successful clinical deployment. As such, the evaluation should focus on clinical relevance, using metrics that assess patient health outcomes. These include task-specific metrics such as lesion detection [75], subjective clinician evaluation through reader studies [76], and cluster randomized clinical trials to evaluate the broader impact on patient care processes [77]. However, these approaches face additional challenges, such as difficulties in conducting double-blind studies and accounting for indirect effects on the delivery of care.

## MRI acquisition

### What is an MRI sequence?

The acquisition process is controlled by the MRI pulse sequence. An MRI sequence defines external magnetic fields, time delays, and signal acquisition periods to manipulate magnetization, so that encoded signals can be acquired to generate images. This control is made possible by the response of the magnetization with respect to external magnetic fields, given by the basic Bloch equation [78]

1 $$\begin{aligned} \dot{\vec {M}} = \gamma \vec {M} \times \vec {B} = \gamma \vec {M} \times \begin{pmatrix} B_{1x} \\ B_{1y} \\ B_0 + B_G\end{pmatrix}, \end{aligned}$$

with the external magnetic field $\vec {B}$ realized by the typical hardware with the components as

- $B_0$: Static field along $z$-axis, drives Larmor precession ($\omega _0 = \gamma B_0$);
- $B_1$: RF field in the xy-plane, drives excitation, refocusing, and preparation;
- $B_G$: Gradient field $\vec {G}(t)\cdot \vec {r}$, drives spatial encoding, spoiling, and diffusion weighting.

In the rotating frame of reference, the Larmor precession with $\omega _0$ is removed and only the influence of $B_1$, $B_G$, and smaller $B_0$ inhomogeneities remain. The full Bloch equations also include relaxations, namely **T1**, the longitudinal recovery time, and **T2**, the transverse decay time, which lead to a time-dependent change of the magnetization vector length, and strong dependence on timing. Moreover, magnetization transfer (MT), diffusion, and other dynamic effects can be incorporated in extended equations sets [79, 80].

An MRI pulse sequence steers the magnetization dynamic using the response to external magnetic fields given by the Bloch equation.

Thus, to steer the magnetization dynamics, which is the actual task of MRI sequences, we have to control dynamic RF fields $B_1$, gradient fields $B_G$, and delays between these events affecting T1 and T2 relaxation, as well as phase accumulation and dephasing effects (e.g., $\Delta B_0, T_2^*$). Finally, once magnetization is properly excited and encoded, we have to define the signal acquisition, which completes the **MRI sequence building blocks**:

1. **RF events**: Dynamic RF events with time-varying amplitude $|B_1(t)|$, frequency $\Delta \omega _{\textrm{RF}}(t)$, and phase $\phi _{\textrm{RF}}(t)$. An emerging parameter is the excitation flip angle ($\alpha$).
2. **Gradient events**: Define spatial encoding via dynamic gradients ($G_x(t), G_y(t), G_z(t)$) leading to the emerging gradient moments $\vec {g}=\int _t{\vec {G}(t)}{dt}$.
3. **Time delays**: Control dephasing, rephasing, and relaxation ($\Delta t$).
4. **ADC events**: Toggle signal acquisition and set ADC phase.

#### MRI sequence definition

**Fig. 2:**
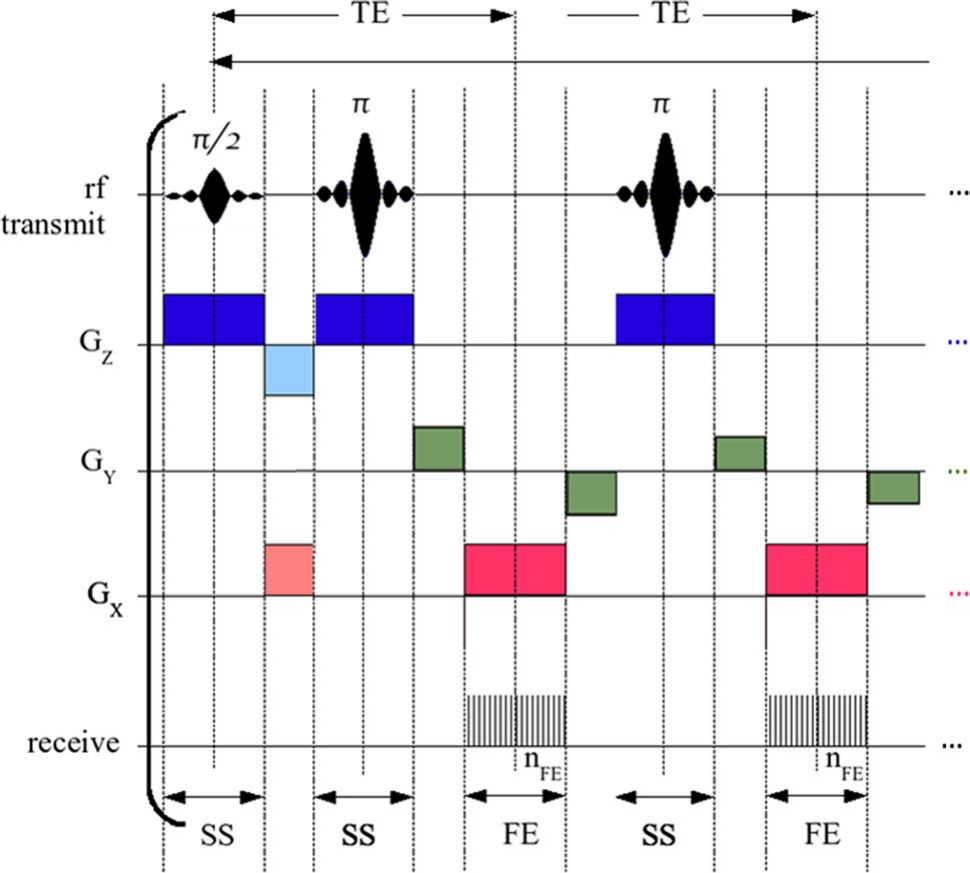
A TSE sequence diagram adapted from Wikipedia (https://commons.wikimedia.org/wiki/File:MRI_2DFT_SE_PulseSequence.png). All building blocks are depicted: RF events, gradient events for slice selection, phase encoding and readout encoding, as well as signal acquisition events ADC. Find the Pulseq-re-implementation of this sequence scheme in Fig. 3

**Fig. 3:**
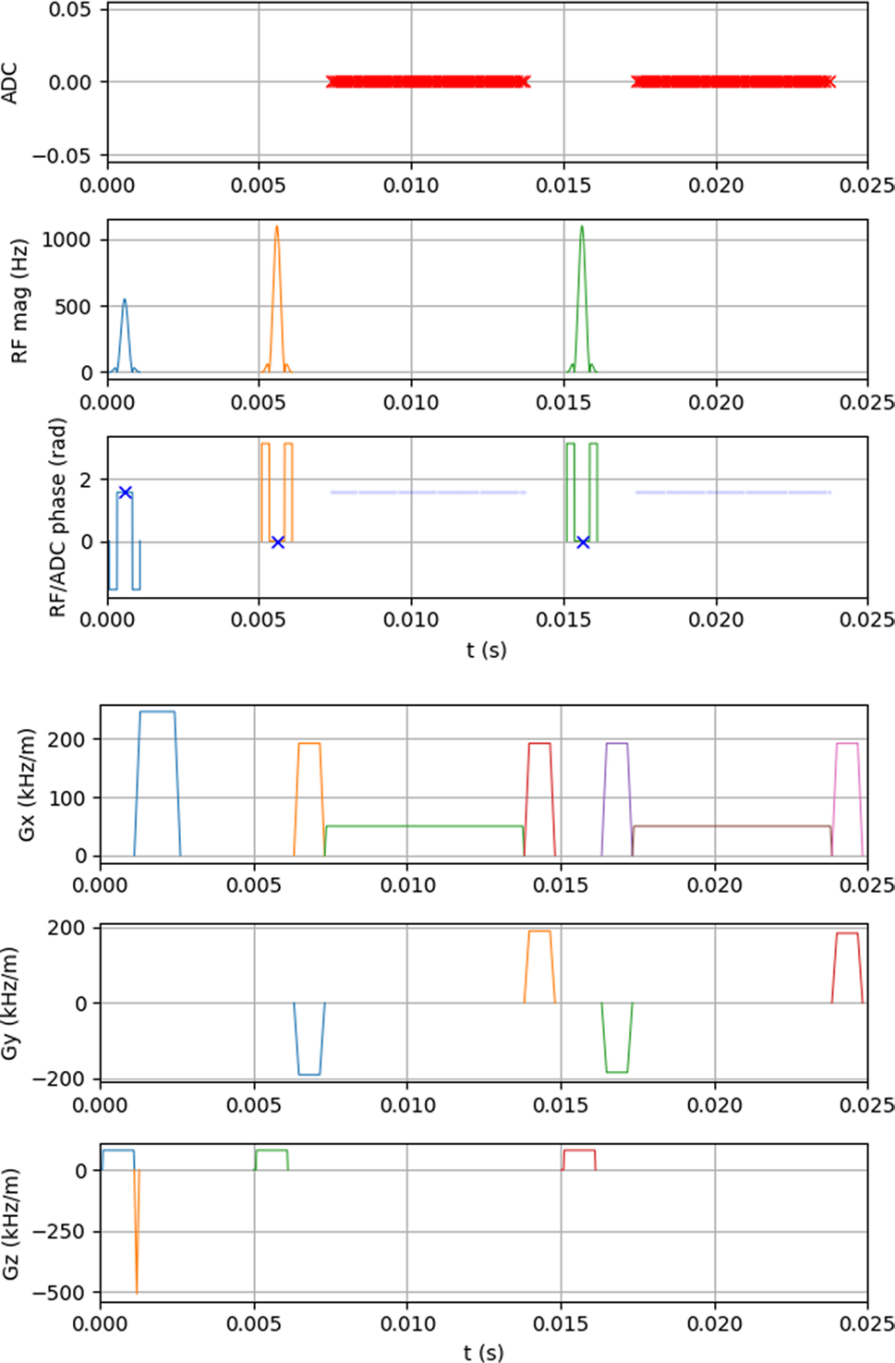
Pulseq-re-implementation of the TSE sequence scheme in Fig. 2. The MRI sequence plot of the Pulseq sequence definition is plotted for a similar time period. In contrast to Fig. 2, we see RF and analog-to-digital converter (ADC) phases, gradient ramps and spoilers, and units of the quantitative plots. Moreover, this is just a visualization of the underlying definition, which can be explored interactively for the complete sequence and arbitrary time ranges at https://github.com/MRsources/Article_MAGMA24_RMRI

An MRI pulse sequence is a particular time scheme of the above-defined building blocks: RF events, gradient events, and receive or ADC events. A visualization of an MRI sequence is the sequence diagram or scheme, as depicted for a turbo spin echo (TSE) sequence in Fig. 2. While this is a compact form of depiction, and schemes are helpful to clarify the idea, they are not always complete in terms of reproducibility. A different type of sequence depiction is shown in Fig. 3, which shows a screenshot of an exact plot of a TSE sequence definition implemented in the generic open-source sequence development framework Pulseq [40].

- An **MRI sequence scheme** gives an implicit recipe of the implementation.
- An **MRI sequence definition** reflects the exact implementation of the sequence, i.e., the full dynamic information of RF, gradient, and ADC events.

**Fig. 4:**
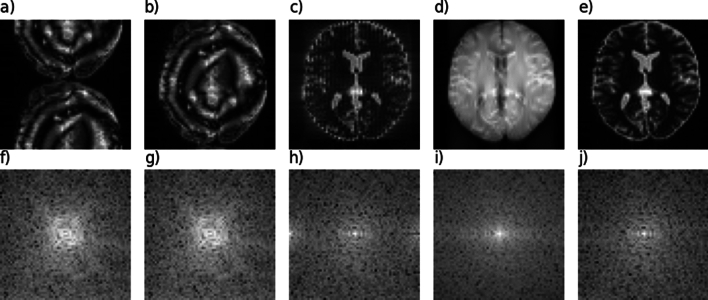
Simulation results of five attempts of recreating the TSE sequence scheme of Fig. 2 in Pulseq. **a** Phases of RF and ADC are not defined; the assumption RF phases = ADC phases = 0 leads to an N/2 shift and artifacts. This is the unstable realization of a TSE sequence. The N/2 shift can be removed by an alternating ADC phase as shown in **b**, but the artifacts remain. In **c**, the correct CPMG $90^{\circ }$ phase shift of the excitation pulse is implemented, but no crusher gradients are shown in Fig. 2, so they were missing leading to a high-frequency FID contribution visible in the k-space plot (**h**) and leading to a high-frequency artifact. With crusher gradients added, this artifact is eliminated in **d**, but here a centric reordering was assumed leading to a very different contrast from the “correct” implementation in **e**, which corresponds to Fig. 3. Each of the simulations can be reproduced from https://github.com/MRsources/Article_MAGMA24_RMRI

While the TSE sequence and its specific implementations [81] are well known, let us try—as a toy example for more complex cases—to reproduce the sequence described in Fig. 2 only from the sequence scheme. Attempting this we realize the following issues:

1. The RF pulse phase is not given, potentially leading to an unstable TSE version. See Fig. 4a.
2. The ADC phase is not defined; an alternating ADC phase removes the N/2 shift, but unstable TSE artifacts remain (Fig. 4b). Additionally, the bandwidth of the readout remains unclear, and it is not specified whether oversampling was used, which, if not employed, could lead to fold-overs artifacts.
3. The gradients have vertical slopes, but the actual slew rates or the time needed to achieve the gradients’ full amplitudes are not provided. The exact shape can also be important when diffusion processes are studied, or when eddy currents are a problem.
4. More importantly, crusher gradients are not depicted. Without these, the sequence will be prone to B1 inhomogeneities, see Fig. 4c. In a TSE sequence, crusher gradients are applied before and after the refocusing pulse to suppress unwanted transverse magnetization, in this case from free induction decay (FID) signals, which otherwise can lead to the observed high frequency artifact.
5. The RF pulse appears to resemble a three-lobe sinc, but its apodization is not specified. Also its slice selection thickness cannot be extracted from the sequence scheme alone, as well as its exact duration, which turns out to be important later.
6. The actual phase-encode ordering can only be guessed; it seems that the phase encoding gradient amplitudes are decreasing. Reordering will induce potential reordering-dependent artifacts, as well as the effective echo time, leading to different contrast, see Figs. 4d,e.

Thus, already an established sequence can be hard to reproduce even qualitatively from a limited description, and novel developments are probably even harder to reproduce. With this in mind, we suggest the following recipe to describe MRI sequences in a reproducible manner.

**Recipe for reproducible publication of MRI sequences:**

- Provide the exact implementation, i.e., $\vec {G}(t)$, RF amplitude(t) and RF phase(t), and ADC(t) with phase, in a complete sequence definition standard.
- Use versioning tools and online repositories (discussed in “General tools for reproducibility”) to provide generating code and/or the sequence definition files along with the publication.

Although using an exact pulse sequence definition substantially reduces variability across different scanners, it is important to note that these frameworks cannot control every aspect of the acquisition process. For example, several steps may depend on scanner-specific factors, including the following:

1. **Gradient pre-emphasis** may differ across scanners and software versions, potentially influencing the fidelity of the sequence.
2. **B0 and B1 shimming** can vary depending on scanner hardware and setup.
3. **Pulse sequence parameter adjustments**: Vendor-specific optimizations such as slice selection, RF pulse shapes, and timing adjustments that may need to be customized for a specific scanner or software version.
4. **Coil setup and sensitivity calibration** might vary and affect signal-to-noise ratio (SNR) or acceleration performance.
5. **Scanner software or interpreter versions** can affect the actual sequence played out at the scanner.

It should be emphasized that different versions of the chosen framework or vendor-specific interpreters may yield slightly different results. For instance, variations in hardware, software updates, or firmware changes can all influence the final pulse sequence outcome. While these frameworks provide an important level of standardization, it remains crucial to carefully test and verify pulse sequences across the intended platforms to ensure consistent and reproducible results.

#### Real-world flavours

**Fig. 5:**
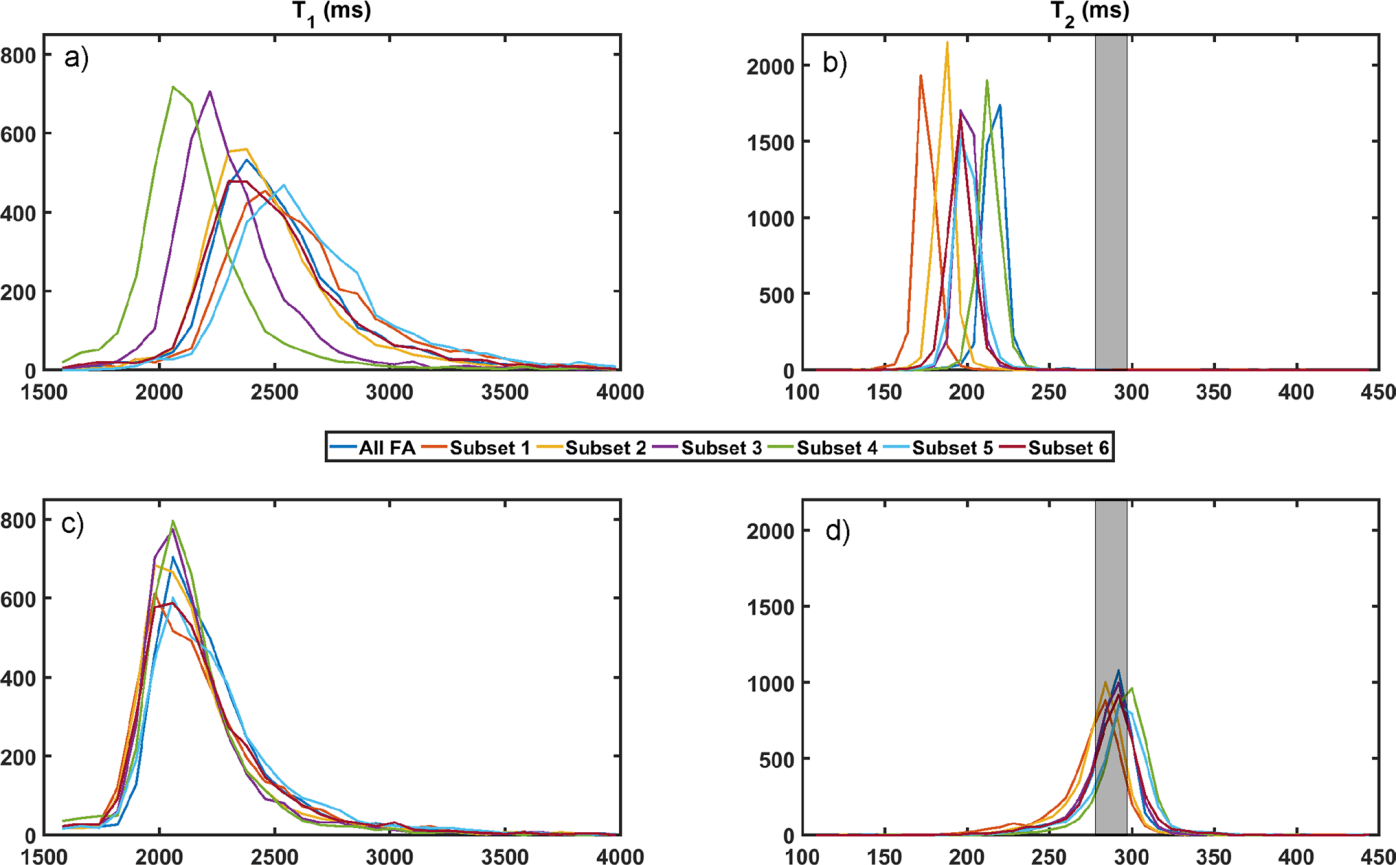
Comparison of $T_1$ and $T_2$ relaxation time distributions without (**a**, **b**) and with (**c**, **d**) considering ($B_{1\textrm{rms}}$) of the RF pulses. The reduction in variability across subsets highlights the significance of exact pulse sequence definition knowledge, so the implicit parameter $B_{1\textrm{rms}}$ is controlled leading to consistent magnetization transfer (MT) effects. Reproduced from [86]

*Flip angle vs. exact pulse definition:* While the RF flip angle, i.e., the time integral of an RF pulse, is often assumed to be its major property, other properties lead to different outcomes, that is:

- The **RF bandwidth** can have a large impact on fat/chemical shift artifacts in the slice direction.
- The **slice profile** can influence SNR, 2D slice cross-talk, and can often affect quantitative results due to a varying flip angle over the slice profile [82, 83]. In a 3D acquisition, a poor slab profile can result in fold-over artifacts as well as a lower flip angle in the peripheral slices.
- **The indirect effect of the root-mean-squared B1** ($B_{1\textrm{rms}}$) on MT effects leads to a large spread of quantitative MRI values, as reported in [84, 85]. While the same flip angles and slice profiles can be generated with various RF and gradient shapes, they might have a different $B_{1\textrm{rms}}$ and thus a different MT effect, which leads to significant differences in, e.g., T1/T2 weighting and mapping as explained in [86]. Knowing the exact sequence definition would allow reproducing the MT effects, and thus arrive at similar relaxation rates. When $B_{1\textrm{rms}}$ is taken into account, major improvements to quantitative estimates can be realized; see Fig. 5.

*Vendor neutrality:* Karakuzu et al. [21] demonstrated that vendor-neutral pulse sequences, implemented using SpinBench and the RTHawk platform (Vista, Inc.) [87], significantly reduced inter-vendor variability in quantitative MRI acquisitions. Standardizing the sequence definition using a common protocol improved reproducibility across different MRI systems. This was also demonstrated in a recent study with 0.55 Tesla systems [88]. This addresses critical challenges in multicenter studies and highlights the importance of reproducibility efforts for reliable clinical and research applications.

*Wrong assumptions in CEST preparation.* In the Bloch–McConnell simulation challenge,^2^ simulations of the same CEST preparation were compared by more than ten different research groups. Unexpectedly, variations above 100% were observed, and could be reduced by simply attempting to achieve reproducible results between groups. One deviation was difficult to find: the 6 ms duration of a crusher gradient at the end of the CEST preparation was assumed to be negligible for the CEST outcome following a T1 dynamic. It turned out that the exact timing definition given in the Pulseq-CEST format [48] resolved the deviation, as after the CEST preparation, the MT and water pool are in non-equilibrium and the short T1 eigenvalue [89] leads to a significant relaxation in that short amount of time. Interestingly, this short T1 eigenvalue is also addressed to be the source of discrepancies in standard T1 mapping [85].

### MRI sequence definition tools

It is recommended to share the complete sequence definition, instead of only an MRI sequence diagram. Pulseq [40], PyPulseq [90], TOPPE [41], GammaStar [42], and SpinBench [87] are example software for defining of a complete pulse sequence’s timing and waveforms, which can be executed on scanners from different vendors and running different software versions. Generating codes or sequence definition files can then be shared online^3^ or as supporting information for a research paper. The mentioned tools are available via:

- **Pulseq**: https://pulseq.github.io/, open source (MIT license), MATLAB.
- **PyPulseq**: https://github.com/imr-framework/pypulseq, open source (AGPLv3 license), Python.
- **TOPPE**: https://github.com/toppeMRI/toppe, open source (MIT license), MATLAB and C++.
- **GammaStar**: https://github.com/gammastar/gammastar, closed source, C++ interface.
- **SpinBench**: https://vista.ai/research-spinbench/, closed source, visual programming interface.

Different realization of an RF pulse, different implementations of the same sequence type, or unexpected effects neglected in the initial design of the experiment can hinder reproducibility, replicability, and ultimately scientific progress.

### Scan process: patients, healthy subjects, phantoms, and simulation

Once the sequence is defined and made interpretable and executable by an MRI scanner, MRI acquisition can proceed. Here, we assume that the scanner reliably implements the defined pulse sequence. To ensure reproducibility, as previously defined, it is essential to control the “input data.” In the context of scanning, these “data” are not purely numerical but represent the object being scanned. Computationally, it might be expressed as a spin system defined by a physical object. True reproducibility (as defined in “Introduction”) can only be achieved under identical conditions: the same scanner, the same pulse sequence, and the same object or subject. In practice, reproducibility tests on patients are rarely performed due to ethical and logistical challenges. Instead, replicability studies—conducted on different patients with the same disease—serve as the cornerstone of clinical validation [91].

Replicability can be tested using multiple healthy subjects, considering age and other healthy variability issues. If not given otherwise by statistical sample size considerations [92], at least ten subjects should be included, matching the status quo [93]. Replicability across different scanners with the same subjects is sometimes assessed and can be achieved by traveling-subject studies [94, 95]. Still, even repeated same-subject scans might be affected by different motion, pulsation, breathing patterns, etc. Phantoms or computational models bring us closer to testing actual acquisition.

***MRI phantoms and computational models***

Phantoms play a crucial role in assessing the reproducibility of MRI by providing a stable and standardized reference for evaluating system performance across time, devices, and sites. These artificial models, designed to mimic the physical or anatomical properties of tissues, enable consistent testing of image quality parameters, such as spatial resolution, SNR, and geometric accuracy. Phantoms reduce variability caused by biological factors and patient-specific differences, allowing researchers and clinicians to isolate and address technical inconsistencies in MRI systems. By utilizing phantoms with known characteristics, studies can more effectively quantify inter- and intra-scanner reliability, validate new imaging protocols, and ensure compliance with quality control standards. There are multiple vendors on the market providing high-quality reproducible and even quantitative phantoms, such as the ISMRM/NIST phantom [24]. But also simple recipes like agarose phantoms [96–98] with standard contrast agents can help, and can be fully open source. In both cases, additional parameters like buffer concentration or type, salinity, pH, and temperature of the phantom can be important to report. Fresh chicken eggs can also serve as a simple but quite reproducible biological phantom [99].

With scanner hardware and physical phantoms, still uncertainties remain, as both are never fully controlled and not fully computational. The missing link that can also turn the acquisition into a pure computational problem is **MRI simulation**. Here, the input data, i.e., tissue parameter maps, can be exactly defined and reproduced. Interestingly, until now, there is no comprehensive and quantitative comparison of the different available simulation tools [43–49]. Now that available sequence definition standards are available, enabling such comparisons, we highly encourage this enterprise. With cross-validated simulations, the chain from tissue parameter maps, sequence definition, and reconstruction, and even parametric mapping can be defined and tested in a reproducible manner. One such chain, including the Pulseq defintion [40], the JEMRIS simulation [43], and BART reconstruction [51], is given by Veldmann et al. [55]. Another prototype using PyPulseq [40], the MR-zero simulation [49], and MRpro [100] or BART reconstruction [51] is given within this article.^4^

## MRI reconstruction

### What is MRI reconstruction?

The MRI signal acquired by the scanner is the voltage induced by the precession of the transversal magnetization due to Faraday’s law of induction in the receive coils. Representing the transversal components of the magnetization $\vec {M}$ as a complex number $M_{xy}=M_x+iM_y$, and neglecting off-resonance and relaxation, the complex-valued raw signal (after quadrature demodulation) of the *i*th receive coil, $s_i(t)$, reads

2 $$\begin{aligned} s_i(\vec {k}(t))= & \int _{\mathbb {R}^3} \textrm{d}^3 \vec {r}\, M_{xy}(\vec {r}) c_i(\vec {r}) \exp \left( -2\pi i \vec {k}(t)\cdot \vec {r}\right) \nonumber \\ & + \eta _i(t) \quad \text {with}\quad \vec {k}(t)=\int _0^t \textrm{d} \tau \, \frac{\gamma }{2\pi } \vec {G}(\tau ). \end{aligned}$$

In words, the acquired signal is the Fourier transform of the transversal magnetization weighted by the coil sensitivity maps $c_i(\vec {r})$. The measurement noise $\eta$ is normally distributed and can be assumed to be uncorrelated after a prewhitening step. The spatial frequencies $\vec {k}(t)$ at which the signal is evaluated is called the k-space trajectory, and it is controlled by the gradient fields. The task of MRI reconstruction is to recover the transversal magnetization, i.e., the image, from the acquired signal, which can be mathematically formulated as an inverse problem. The simplest reconstruction is achieved by sampling the signal on a Cartesian grid satisfying the Nyquist frequency, such that the image can be directly recovered by an inverse discrete Fourier transform.

To accelerate the MRI acquisition, the signal is often sampled below the Nyquist rate, resulting in aliasing of the reconstructed image if not compensated for during the reconstruction process. While it is possible in some cases to undo the aliasing from a single coil [101], the most common approach is to exploit redundant information across distinct receive coils to resolve aliasing through parallel imaging. k-Space-based methods such as SMASH [102] and GRAPPA [103] compose a fully sampled k-space by interpolating sampled points to the missing k-space points. Image based methods such as SENSE [104] explicitly use coil sensitivities to unfold aliasing in the image domain.

***Parallel imaging and compressed sensing (PICS)***

A common approach is to represent the reconstruction as the regularized least-squares cost function [105, 106]

3 $$\begin{aligned} x^* = \arg \min _x \Vert y - Ax \Vert ^2_2 + R(x)\, , \end{aligned}$$

where *x* and *y* are vectors containing the image and the k-space data, respectively. $A=\mathcal {PFC}$ is the linear operator mapping the image to the k-space data by multiplying it with the $\mathcal {C}$oil sensitivities, followed by $\mathcal {F}$ourier transform and projection to the (potentially non-Cartesian) sampling $\mathcal {P}$attern. *R* is a regularization term penalizing unlikely images. By selecting sparsity promoting regularization, e.g., $\ell _1$-Wavelet or total variation, and a randomized sampling pattern, (3) yields compressed sensing (CS) reconstructions [105, 107]. Under a Bayesian lens, (3) can be viewed as the maximum a posteriori (MAP) estimate, i.e., $R(x) = -\log p(x)$, where *p* is the probability density function indicating the prior likelihood of a particular image.

For any nontrivial choice of sampling pattern, solving (3) involves the implementation of an iterative algorithm such as the Conjugate Gradient method [108], proximal algorithms [109], or generalizations, e.g., through plug-and-play methods [110]. In addition to the regularization parameter, these algorithms introduce additional hyperparameters like the step size, the number of iterations, or early stopping rules, which need to be reported to ensure reproducibility.

***Deep learning-based reconstruction***

Deep learning has transformed MRI reconstruction by replacing traditional hand-crafted regularization terms with data-driven approaches that learn statistical properties of MRI images from training datasets. These methods enable faster and more accurate reconstructions than classical approaches, hence they have had a broad impact across diverse applications. See [111] for a recent review. At the heart of most deep learning reconstructions is the training over a large corpus of data (though scan-specific training is also possible), including tuning of specific reconstruction parameters (such as the neural network architecture) using a validation dataset. Reconstruction performance is then evaluated using a hold-out test set not used during training. In some cases, publicly available data will enumerate the train, validation, and test splits [112], though publications using the data may create new splits, for example to reduce the dataset size for computation purposes or to constrain the types of scans considered (T1 vs. T2 weighting). To achieve reproducible deep learning models, the datasets, their preprocessing, and the data splits need to be reported along with the model architecture. Sharing both is also part of the Open Source AI Definition [113].

### MRI reconstruction definition

While often the design and optimization of (3) for reconstruction is described in detail, it should be noted that this is just one part of the reconstruction pipeline from the raw k-space data to the final image, where a typical complete reconstruction pipeline usually consists of the following steps:

1. Noise prewhitening, i.e., removing the correlation of noise from different receive coils;
2. Normalization of k-space data, for example based on noise level or maximum image magnitude;
3. Possible k-space corrections (gradient delays etc.);
4. Coil compression for reduced reconstruction complexity;
5. Estimation of coil sensitivities;
6. PICS or deep learning reconstruction as described in “What is MRI reconstruction?”;
7. Image processing such as distortion corrections, filtering, or denoising;
8. Potential estimation of quantitative parameter maps;
9. Windowing and interpolation to generate the final image printed in the paper.

In the context of deep learning, all steps of data processing also apply for the generation of training data, e.g., pairs of subsampled k-space data, coil sensitivity maps, and reference reconstructions.

An *MRI reconstruction definition* is the software code that takes the raw k-space from the scanner and produces the reconstructed image or downstream output.

Recipe for reproducibility of MRI reconstruction:

- Provide the complete software code that reflects the MRI reconstruction definition, including the complete list of software versions that were used in the computational environment (e.g., by a *yaml* or JSON file).
- Best practice: Provide the data that were processed by the reconstruction code in a vendor-neutral format. Second best: provide a subset or example data.
- Best practice: Provide unit tests that check for correctness of the reconstruction output. Second best: provide the expected result.

### MRI reconstruction tools

In principle, reproducible MRI reconstruction can be achieved with proprietary, closed-source software if the software versions are accurately specified. Nevertheless, it is recommended to avoid proprietary software as its use restricts reproducibility to those sites having access to the corresponding licenses, and it introduces black boxes. Fortunately, many mature open-source reconstruction toolboxes, such as BART [51], Gadgetron [50], MiRT [114], MRIReco.jl [52], MRpro [115], and SigPy [116], exist which allow for fully transparent open-source reconstruction pipelines. Those toolboxes usually provide implementations of algorithms for estimation of coil sensitivity maps, non-uniform FFTs, and iterative algorithms to solve (3). The mentioned tools are available via the following:

- **BART**: https://mrirecon.github.io/bart, open source (BSD), C (commandline tools and bindings for Python and Matlab available)
- **Gadgetron**: https://github.com/gadgetron/gadgetron, open source (modified MIT), C++
- **MiRT**: https://github.com/JeffFessler/mirt, open source (MIT), Matlab
- **MRIRecon.jl**: https://github.com/MagneticResonanceImaging/MRIReco.jl, open source (MIT), Julia
- **MRpro**: https://github.com/PTB-MR/mrpro, open source (Apache 2.0), Python
- **SigPy**: https://github.com/mikgroup/sigpy, open source (BSD), Python.

### (Not) reproducible reconstruction: an example

Given the k-space data, MRI reconstruction is in principle deterministic and hence reproducible as, in contrast to acquisitions, no unknown factors such as system imperfections or noise may vary the outcome from run to run. Nevertheless, without the availability of code, even very detailed descriptions of the reconstruction may not lead to reproducible reconstructions.

To demonstrate this, we performed parallel imaging and compressed sensing (PICS) reconstruction in BART (version v0.9.00) of a fully sampled 2D TSE brain scan (Siemens Prisma 3T, 32 coil array) collected on a volunteer with institutional board approval and informed consent. Scan parameters include: $220\times 220 \, \textrm{mm}^2$ field of view, $0.5\times 0.5\, \textrm{mm}^2$ resolution; 4 mm slice thickness, turbo factor = 15. As a fully sampled reference, we inverse Fourier transformed the k-space data, coil combined them using coil sensitivities estimated with ESPIRiT [117], and computed the magnitude. We retrospectively subsampled the fully sampled k-space data by multiplying it with a fourfold random Cartesian sampling pattern with 24 autocalibration lines. We repeated the estimation of coil sensitivities on the subsampled, coil-compressed k-space data, removed the frequency oversampling, and reconstructed it using $\ell _1$-Wavelet regularization with a regularization parameter of $\lambda =0.001$.

**Fig. 6:**
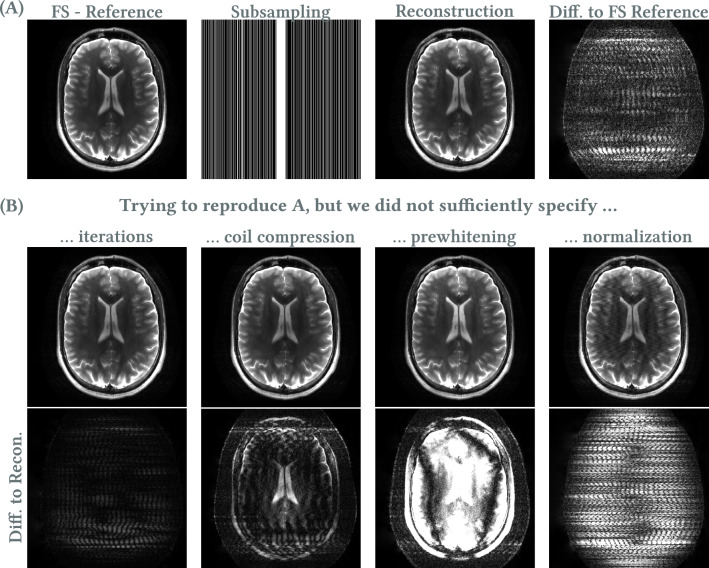
Variation of $\ell _1$-wavelet reconstruction due to changes in not reported parameters. Differences due to variations in the parameter are in the same order as differences between the fully sampled reference reconstruction and the default reconstruction. Difference maps are scaled by a factor of 20

While this description of the reconstruction is not very detailed, it is typical for scientific reports if the focus is not on the reconstruction or the PICS method is only a comparing method for a proposed method. In Fig. 6A, we present our reconstruction and its difference with the fully sampled reference. We then show several reproducibility failures due to unreported properties leading to errors of similar order to the difference between the “correct” reconstruction and the fully sampled reference as shown in Fig. 6B. Namely, we did not specify the following:

1. the number of iterations: we used 50 instead of 300 here;
2. the number of virtual coils: we changed it from 12 to 8 here;
3. prewhitening of the k-space data: we omitted it here;
4. the normalization of k-space data: we changed it from BART’s default scaling to an arbitrary scaling, which effectively scaled the regularization parameter.

Depending on the scope of a manuscript, reporting every parameter which may be tuned is often not possible. Nevertheless, sharing of code together with raw data and versions of used software makes the experiments reproducible.

### Real-world flavours

**Fig. 7:**
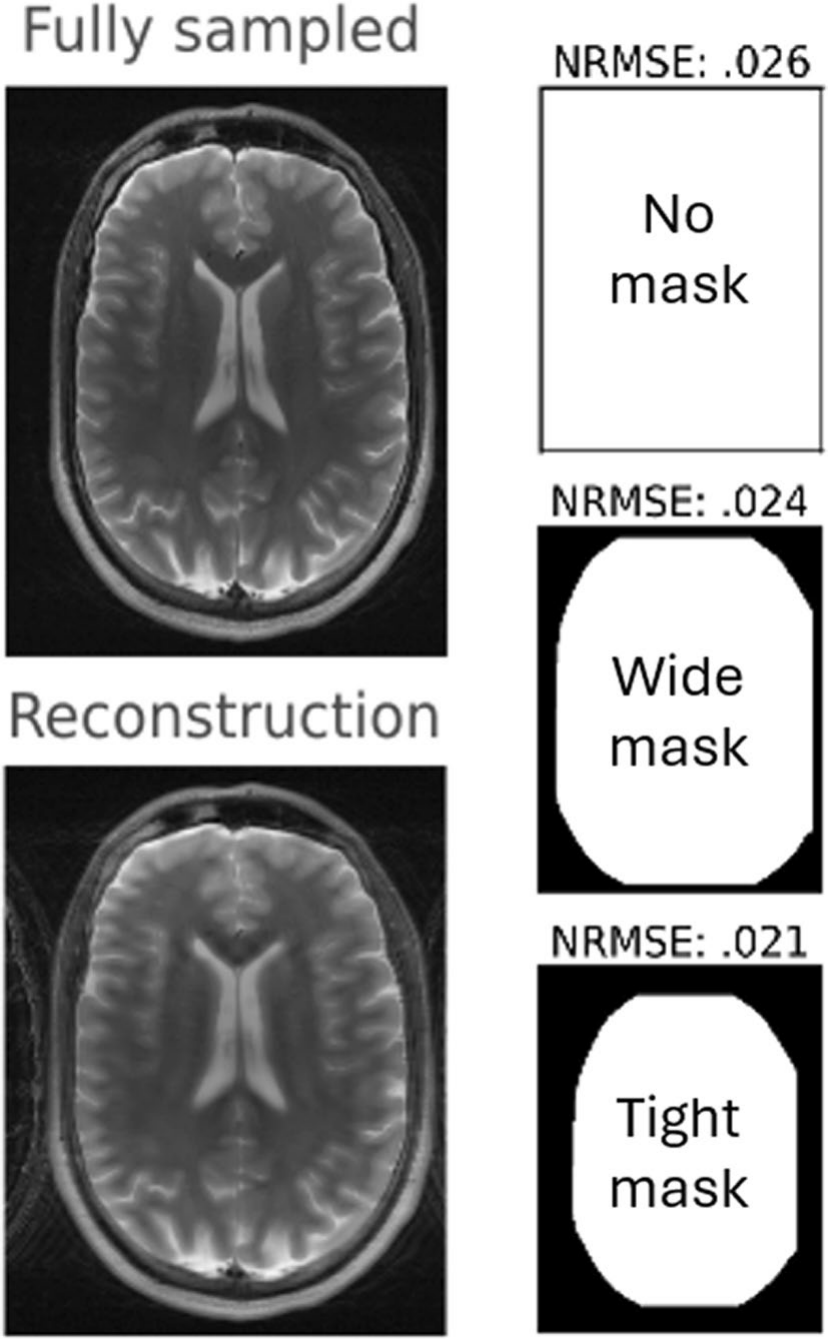
Influence of the masking strategy on the image quality metric. The normalized root-mean-square error (NRMSE) was computed with three strategies: no mask, a wide mask, and a tight mask. Notice that although the reconstructed image does not change, using different masks yields substantially different NRMSE values

*Challenges with reference-based error metrics*. Reference-based error metrics, commonly used to evaluate MRI reconstruction methods, can lead to unfair or inconsistent assessments [29] if not fully specified. A key issue is the choice of the reference image itself, as mismatches between the reference and reconstructed images can provide misleading evaluations. For example, training unrolled networks to reconstruct complex-valued multi-coil data while evaluating their results against magnitude-only root sum-of-squares data can lead to artificially low scores, even when the reconstructed images are of high visual quality [118]. Additionally, reference-based metrics are highly sensitive to noise, alignment, and the application of masks during computation. To illustrate the latter, we conducted an experiment using reference and reconstructed brain images containing background noise. We evaluated the normalized root-mean-square error (NRMSE) without a mask, where it was 0.26; using a wide mask, where the NRMSE reduced to 0.24; and using a tighter mask that excluded most of the background, where the NRMSE further reduced to 0.21 (Fig. 7). These results highlight how differences in masking can lead to inconsistent evaluations of reconstruction methods.

*Challenges with hidden noise*. Another challenge with the use of reference-based image quality metrics is that their computation ideally requires a clean, noise-free reference image. However, in MRI, acquiring such images is infeasible due to thermal noise [119, 120]. Therefore, even images acquired with Nyquist-rate sampling contain some level of noise. However, because reference-based quality metrics treat such noise as a desired target, the noise can affect the evaluation of reconstruction methods. For example, in parallel imaging, noise in the reference data could bias conventional performance metrics such as SSIM and lead to suboptimal method rankings [119]. Furthermore, in low-field MRI, where SNR is commonly very low, using reference images obtained from a small number of repetitions (a.k.a. small NEX) can distort error metrics compared to the use of images obtained with many repetitions, and hence misrepresent reconstruction quality [121].

*Challenges with retrospective subsampling*. Retrospective subsampling, commonly used in developing reconstruction algorithms, introduces challenges that can bias results and hinder reproducibility. When k-space data synthesized from reconstructed images, such as those from public repositories, are subsampled, hidden preprocessing pipelines (e.g., lossy compression) often enhance data sparsity. This artificially boosts algorithm performance during evaluation, leading to overly optimistic results that fail to generalize to raw data [29]. Another challenge emerges in transient sequences, such as MP-RAGE or FLAIR, where signal evolution depends on the history of k-space acquisitions itself. As demonstrated in recent studies [122, 123], retrospective subsampling of these sequences fails to account for altered transient magnetization dynamics. For example, subsampling k-space lines effectively removes excitation or refocusing pulses from the prospective sequence, leading to k-space data corresponding to sharper reconstructed images than those of the original sequence. If not performed with care, this approach can cause data domain shifts between retrospectively subsampled and prospectively acquired data. To raise awareness of such issues, the publication of misleading results due to inadequate or un-careful retrospective subsampling has been dubbed **“data crimes”** [29].

## General tools for reproducibility

### Software and versioning

Good practices for code sharing and version control are essential to reproducible research. Below, we share a recipe for code management and dissemination, as well as list example publications that achieve (or nearly-achieve) full execution of the recipe.

Recipe for code management and dissemination:

- **Use version control systems.** Maintain code and data on a version-controlled system (such as Git) and host the repository on public platforms (like GitHub, GitLab). Include relevant license information to clarify usage rights. Zenodo [124] can be used to tag a version of software with a DOI and guarantees long-term archiving of this version.
- **Add a license.** Permissive licenses, such as the MIT license, are preferable for their minimal restrictions and compatibility with commercial use.
- **Include a README file.** The README should be a quick start guide to help users begin with the code, including project goals, usage instructions, and file organization.
- **Provide installation instructions.** Add an installation instructions page with all dependencies and their versions listed. Provide troubleshooting tips for common issues. Use tools like Docker to encapsulate the environment.
- **Create scripts to reproduce figures.** Each figure presented in the paper should be accompanied by a standalone script that can independently reproduce it.
- **Implement unit tests.** Provide a test suite that validates the integrity of key functions and workflows.
- **Provide a citation to your work!** This makes it easy for end-users to find and cite the corresponding paper from the code repository.

### The incomplete menu of reproducible research tools

**Fig. 8:**
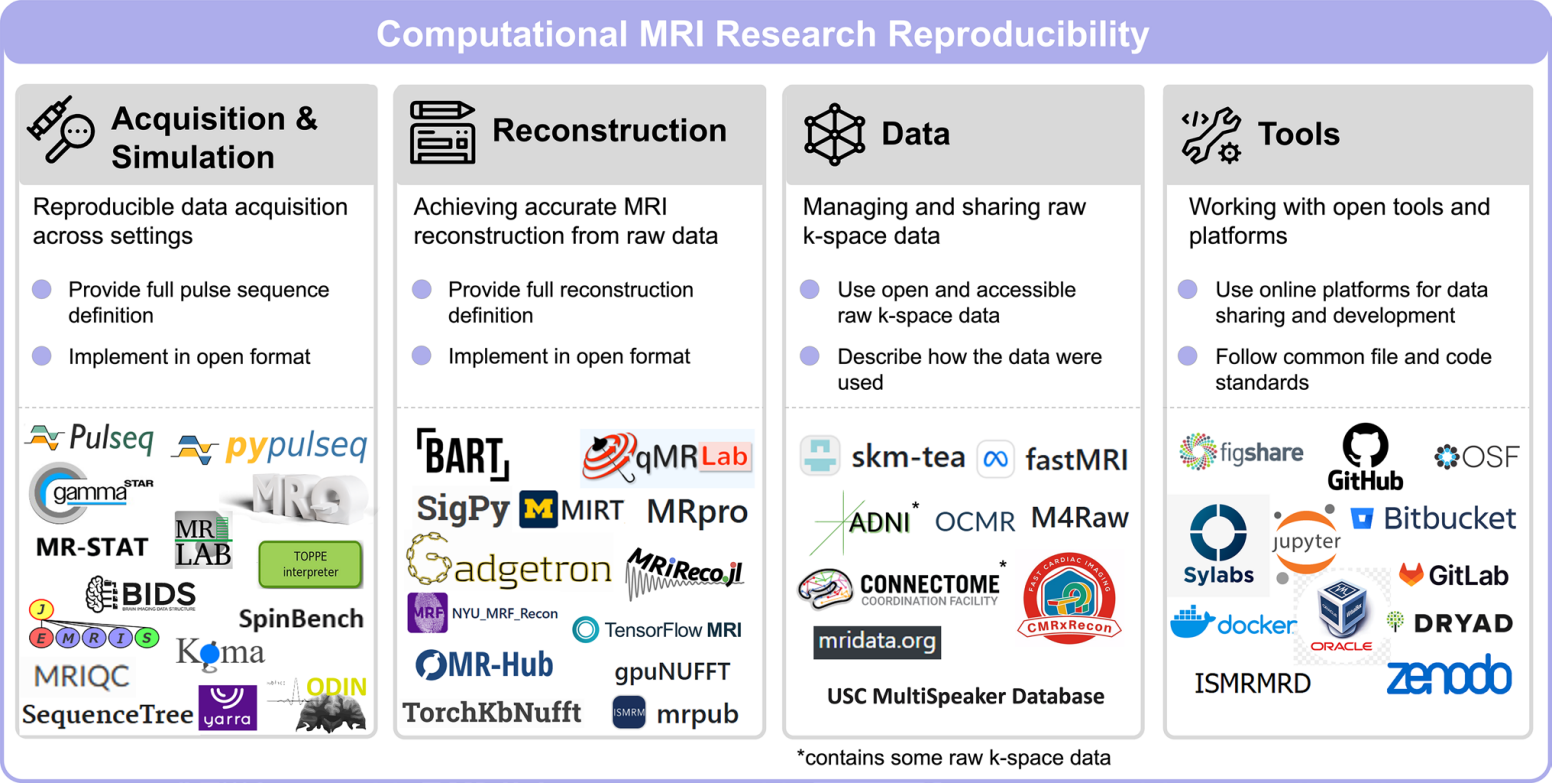
An incomplete collection of reproducible software for computational MRI research, divided into four categories: acquisition + simulation, reconstruction, data, and tools

We finish this article with a list of computational MRI and reproducible research tools that are available online, collected in Fig. 8. While this list is by no means comprehensive, it provides a sampling of the rich reproducible MRI research environment.

We also point the readers to recent papers that helped inspire the creation of the recipes, and thus serve as references: Deep, Deep Learning with BART^5^ [125]; Pulseq^6^ [40]; Open-source MRI chain^7^ [55].

## Conclusion

In this article, we reviewed MRI acquisition and reconstruction in the context of reproducible research. We provided guidelines for creating reproducible research, both explicitly through recipes, and implicitly by sharing code and data to reproduce the figures in the paper. Like other computational sciences, computational MRI is best served on a reproducible platter, and we encourage the community to invest energy and resources to maintain a thriving reproducible ecosystem. To make all that we know reproducible is only the foundation—the starter—for the main course: exploring the novel and unknown. Bon appétit!

## Data Availability

Code to reproduce the results in the figures corresponding to computational experiments is provided in https://github.com/MRsources/Article_MAGMA24_RMRI. Corresponding data are provided under doi: 10.5281/zenodo.14497769.

## Footnotes

1 https://github.com/MRsources/Article_MAGMA24_RMRI/issues.

2 https://github.com/pulseq-cest/BMsim_challenge.

3 https://github.com/MRsources/Article_MAGMA24_RMRI.

4 https://github.com/MRsources/Article_MAGMA24_RMRI.

5 https://github.com/mrirecon/deep-deep-learning-with-bart.

6 https://pulseq.github.io.

7 https://github.com/mrphysics-bonn/python-ismrmrd-reco.
